# Health insurance status of cross-border migrant children and the associated factors: a study in a Thai-Myanmar border area

**DOI:** 10.1186/s12913-022-08681-0

**Published:** 2022-10-23

**Authors:** Chalermpol Chamchan, Kanya Apipornchaisakul

**Affiliations:** Institute for Population and Social Research, Mahidol University, Salaya, Phuttamonthon, Nakhon Pathom, Thailand

**Keywords:** Cross-border migrant, Migrant children, Health insurance, Migrant health, Thailand

## Abstract

**Background:**

Although policies of Thailand for migrant health protection are inclusive for all migrant groups, due to existing constraints in practices and policy implementation, many migrant children still lack the protection. This study aimed to assess the health insurance status of children aged 0-14 whose parents were cross-border migrant workers in Thailand, and factors related to the status.

**Methods:**

A Thai-Myanmar border area, being developed as a ‘special economic zone’ by the Thai government, was selected as a study site. With a cross-sectional research design, the study collected primary data in late 2018 by a structured questionnaire from 402 migrant households that contained 803 children. The logistic generalized estimating equation (GEE) technique was applied to examine factors associated with the children’s health insurance status. These included socio-economic factors, migration factors, and health insurance-related factors.

**Results:**

It is found that 83.2% of the migrant children did not have health insurance. Factors associated with the health insurance status included age 12-14 years (Odds ratio (OR) 2.34; 95% confidence interval (CI) 1.23-4.46), having a birth certificate (OR 1.89, 95% CI 1.04-3.45), and plan of the family in the future to remain the child in Thailand (OR 2.37, 95% CI 1.09-5.17). The primary carer’s factors that were important health insurance-related factors included having no legal work permit (OR 4.12, 95% CI 1.88-9.06), having health insurance (OR 8.51, 95% CI 3.93-18.41), little or no ability to communicate in Thai (OR 0.31, 95% CI 0.14-0.66), and understanding the right of migrant children to purchase health insurance (OR 2.57, 95% CI 1.52-4.34).

**Conclusions:**

The findings point to the need for every migrant child to have a birth certificate, diminishing language barriers, and providing education and motivation about the need for health insurance for migrants and their accompanying dependents, especially children. For further studies, it is suggested to include migrant health insurance supply-side factors with qualitative analyses to understand how all the factors interactively determine the health insurance status of migrant children.

**Supplementary Information:**

The online version contains supplementary material available at 10.1186/s12913-022-08681-0.

## Background

Thailand is a hub for cross-border migration for many working-age migrants and their accompanying dependents, especially from Myanmar, Laos, and Cambodia [1]. Given the many unofficial border crossings, many of these migrants are undocumented [2]. The 2015 estimate of the migrant population of the three nationalities in Thailand was approximately 4.6 million (including an estimated one million accompanying dependents) [3].

Approval for work in Thailand to cross-border migrants does not apply to the residence permit of accompanying child dependents. Thus, children who accompany their migrant parents (or are born and raised in Thailand) could be expected to be none or at a small number. According to the 2004 Registration of migrant workers’ dependents (age under 15 years), the number of registrants in that year was 93,062 [4, 5]. Despite this official number, the actual number of migrant children at any time is expected to be 2-3 times higher, or about 200,000-350,000. However, this rough estimate lacks any empirical data to substantiate the accuracy [5–7]. UNICEF estimated that, in 2019, Thailand had approximately 3.6 million migrants, of whom 14% were children under 18 years, or approximately 500,000 people [8].

In the past, the Thai government did not have a clear policy relating to the minor dependents who accompanied their migrant worker relatives across the border into Thailand. The registration campaigns were periodic and merely in response to Thai Cabinet Resolutions and not part of a systematic program of counting [9]. Most of the studies on the migrant population in Thailand remain focused on the working-age group because that is the majority and has implications for the economic productivity of the nation. As a result, there is a dearth of studies and statistics on migrant children in Thailand at any given time, including basic information on the distribution, living arrangements, health status, and health problems, and schooling of these vulnerable individuals. Given the undocumented status of many of these children and their parents, these youth are at heightened risk of being trafficked into child labor or exploited and victimized in other ways [5, 7, 10].

Thailand's health insurance options for the migrant population seek to be inclusive and accessible. The Ministry of Public Health’s (MOPH) healthcare facilities are allowed to sell health insurance cards under the Migrant Health Insurance Scheme (MHIS) to migrant workers, including accompanying dependents. The enrollment of dependents is, however, on a voluntary basis and practiced with some variations across health facilities [11]. The cost of an insurance card is 365 baht per year for children under the age of 7 years, and 1,600 baht per year for children aged 7 to 14 years, the same as the cost for migrant adults aged 15 years or older [10]. The package of benefits is equivalent to the universal health insurance benefits of Thai citizens, both in- and out-patient care, as well as health promotion and disease prevention services such as age-appropriate vaccination[10, 12]. There is also a non-governmental health scheme offered by certain NGOs, such as the health insurance card of the Migrant Fund (or M-Fund) that currently operates in Mae Sot (Tak Province), Sa Kaeo Province, and Sangkhlaburi District (Kanchanaburi Province). The M-Fund includes also coverage for dependent children of migrants [13].

The fact remains that many migrant children still lack comprehensive health insurance, and that poses dangerous risks for their families if the child becomes suddenly ill or injured [14]. These children, especially the preschooler or out-of-school, are also deprived of age-specific vaccination, and that is a public health issue not only for the migrant communities themselves but also for the surrounding Thai communities [15, 16].

From previous studies in Thailand, the main reasons for not enrolling in health insurance for migrant workers and their dependents were the semi-voluntary nature of the existing insurance schemes, the delays and lack of clarity in the administration process, and uncooperative support from the employers [17]. At the present, studies on enrollment in the health insurance of migrant children are very few, mainly due to a lack of data and difficulties in accessing this group of the population. A recent study addressed the affordable price of health insurance as a key factor associated with the demand for purchasing insurance for migrant workers’ dependents [18]. Somehow, in the context of children in migrant households, studies in other settings suggested that there seem to be many more factors especially social determinants at the household level and migration-related factors that should be investigated [19, 20]. This study had the objective to assess the situation of health insurance coverage for children of migrants (age 0-14 years), and to analyze the factors that determined health insurance enrollment of the migrant children - including socio-economic factors, migration factors, and health insurance-related factors.

## Methods

### Conceptual framework

From the literature review [19–22], the salient factors associated with the child's health insurance or enrollment in health insurance, especially in the context of cross-border migration, are primarily household and primary carer factors rather than the personal factors of the child. These are factors influencing the perception of needs and demand for health protection, seeking those protections, and having the ability to pay for the protection. For this research, the variables can be grouped into the following three: socioeconomic factors, migration factors, and health insurance-related (adapted from Levesque, et. al. (2013)) [23]. Each group can be further stratified by household level, and carer and migrant child factors^1^.

The socioeconomic factors include the economic status of the household, sex and age composition, level of education and employment status of household members, and household income [24–29]. Individual child factors include sex, age, and health status (chronic disease/disability) [28, 30]. For migration factors, pertaining to both the household factors and the child's characteristics, factors include immigrant or legal status and work permit statuses of migrant parents, length of stay in the destination country which is related to the level of social integration of the migrant household in the destination country, previous migration experiences or migration history, plans for the length of stay in the country of destination and return to the country of origin [4, 25, 31–34].

Health insurance-related factors refer to variables that enhance access to health insurance for the migrant and accompanying child dependents. Based on previous studies [4, 15, 27, 35], these factors include access to relevant information to create a foundation of knowledge and favorable attitudes toward the right to and the importance of health insurance, and how to access it. Other factors include language ability and the health insurance status of household members, especially the primary carers. To fully exercise the right to health insurance and other protections, it is essential for the migrant child to have valid identity documents in the destination country (e.g., birth registration) [7, 36].

For this study, variables in the analysis of associated factors with the health insurance status of migrant children were summarized in Table 1.

**Table 1:** Variables in the analysis model and operational definitions

**Independent variables**			**Dependent variable**
	**Variable**	**Operational definition**	
***Socio-economic factors***	Sex (MC)	sex of the migrant child (Boy, Girl)	Health insurance status of the child (0= not have; 1 = have)
	Age (MC)	age of the migrant child (early childhood from 0-5 years, primary-school-age from 6-11 years, and junior-high-school-age from 12-14 years)	
	HH income per head	monthly income per capita of the migrant household (under 1,000 baht, 1,000-1,999 baht, 2,000-2,999 baht, and 3,000 baht or more)	
	Number of children in HH	Number of migrant children aged under 15 years in the household (1, 2, 3, or 4 or more).	
***Migration factors***	Birthplace (MC)	birthplace of the migrant child (Thailand, not in Thailand)	
	Length of stay in Thailand (HH)	length of stay in Thailand of the household (0-4 years, 5-9 years, 10-14 years, 15-19 years, and 20 years or more)	
	Return plan to home country (HH)	plan to return to the home country of the household (within the next 2 years, within 2-5 years, no plan to return, and undecided)	
	Future plan for the child (HH)	future plan for the migrant child(ren) in the household after reaching age 15 (not remaining in Thailand, remaining in Thailand)	
	Work permit (PC)	possession of a work permit of the primary carer of the migrant child (Yes, No)	
***Health insurance-related factors***	Birth registration (MC)	possession of a birth certificate for the migrant child(ren) (Yes, No)	
	Health insurance status (PC)	health insurance coverage for the primary carer (Yes, No)	
	Thai language ability (PC)	Thai language ability of the primary carer (very good, good, moderate, weak, none at all)	
	Knowledge of MC’s right to health insurance (PC)	knowledge (of the primary carer) of the migrant child’s right to health insurance (to buy or enroll with a health insurance scheme) regardless of documentation status (know, not know)	

Note: The variables were measured at the household (*HH* Household, *PC* Primary carer in the household) and individual (*MC* Migrant children) levels

### Study design

This is a cross-sectional study using the data from the research project titled “Migrant children population: Child rearing, access to health services and education in Special Economic Zone (SEZ) Mae Sot, Tak Province”. Fieldwork of the research project was conducted by applying quantitative methods to collect data from a sample of cross-border migrant households who had an accompanying child aged 0-14 years. Mae Sot District of Tak Province, which has a large and densely-settled population of cross-border migrants from Myanmar, was selected as the study site. The main reason was that it has some unique socio-economic and cultural factors and has been designated as an area to be developed as a ‘special economic zone’ by the Thai government. That designation serves as a magnet to attract migrants, whose population in Thailand is expected to climb in the future. Data were collected from the primary carer of the child in the household, aged 18 or over, using a structured questionnaire which was administered by trained interviewers. The questions include items on the general characteristics of the household, history of migration, household situation, and specific characteristics of the children and their primary carer.

### Sampling

The field survey team visited five sub-districts in Mae Sot District which have a particularly large and densely-settled population of cross-border migrants. Snowball sampling was employed to obtain the required number of sample households. The reason for using this method of sampling is because many of the families in this area are undocumented (i.e., without immigrant documents or work permits) and, thus, any official listing of resident households is likely to be incomplete or inaccurate. Given the inability to construct a standard sampling frame, the researcher determined that the snowball sampling technique was the most practical method of selecting respondent households. Only families which had been living in the current location for at least three months were included in the survey, and only families with at least one child aged 0-14 (regardless of country of birth) were included.

The reported number of migrant workers with a work permit in Mae Sot in 2018 was nearly 40,000 [37]. The actual number, though, remains unknown but was believed much higher. By using the sample size formula^2^ suggested by Yamane (1967) with a 5% margin of error [38], the desired sample size in the present study was 400 primary sampling units. The formula indicated that a sample size of 400 would be enough to accurately represent the larger population of interest. To ensure sample diversity, the snowball sampling was designed for not more than ten samples recruited from each seeder household (the initial set of households that the research team contacted with assistance from the network of non-governmental organizations working with migrant communities in the study site). Around 50 seeder households, equally 10 households per sub-districted, were recruited and contacted to generate the 400 sample households.

### Data collection

The questionnaire included items on members of the sampled household, including sex, age, employment, possession of identification documents, history of migration, and duration of residence in Thailand. For the family member(s) who was a child aged 0-14, the questionnaire asked about the place of birth, whether the child has a birth certificate, coverage with health insurance, education, and plan for child care until the child reaches the age of 15. There were also questions to the child’s primary carer about his or her knowledge and attitudes about the rights of the child while in Thailand (Supplementary material Additional file 1, Questionnaire).

The field data collection teams included a team supervisor and interviewers who were all bi-lingual in Burmese and Thai languages. Interviewers were trained to ensure full understanding of each question in the questionnaire, and survey etiquette. A total of four survey teams were deployed, and each team supervisor coordinated with the field team leader daily. Data collection was conducted from October to December 2018.

### Data analysis

Binary logistic regression analysis, with the dependent variable being specified as the health insurance status (insured, uninsured) of the child was employed in the data analysis. Since the primary sampling unit in this study was the household, it is possible that some selected households might have more than one child. Thus, that artifact introduces the possibility of an unknown correlation between outcomes of the dependent variable (due to the fact that the household and primary carer factors for these children would be the same). Accordingly, the multivariate analysis applied the generalized estimating equation (GEE) technique which is used for outcome variable that is continuous or dichotomous in which the responses are correlated. That approach should produce more robust findings and more reliable parameter estimates [39]. In the case of this study, household is a cluster variable, while data for each migrant child in the sample is an observation variable as used in the statistical analysis. Also, since the dependent variable in this study (health insurance status) is dichotomous (i.e., binary), the Logistic GEE modeling was conducted in the multivariate analysis.

Three models, using a hierarchical approach, were generated for the analysis. Model 1 examined only the socio-economic factors. Model 2 looked at the socio-economic factors in conjunction with the migration factors. Model 3 entered variables from all three dimensions (socio-economic factors, migration factors, and health insurance-related factors) into the equation. Corrected Quasi Likelihood under Independence Model Criterion (QICC) statistics of the three models, then, were compared to confirm the model that was the most suitable structure.

## Results

The questionnaire surveys were conducted with 402 migrant households which contained 803 migrant children aged 0-14. Overall, about access to health insurance of the migrant child (*n*=803), a large majority (83.2%) did not have health insurance coverage at the time of this study. This means that whenever the child was ill or injured, the family must pay out-of-pocket for clinical care, and must also pay for any health promotion or disease prevention services as needed. Of the 16.8% with health insurance coverage, most (11.7% of the total sample) had non-government health insurance, e.g., from the private sector employer’s scheme as provided to the migrant worker parent, or an NGO’s scheme such as the M-Fund (either as a family package or the child package). A small percentage of migrant children 3.8% and 1.3% were covered by the health insurance card of the MOPH, the MHIS for children aged under 7 years and 7 years and over, respectively.

Table 2 shows the characteristics of migrant children, their households, and their primary carers. It can be seen that the proportion of boys and girls is similar, their median age was 7 years, and most were in the age group 6-11 years. Over three-fourths (76.2%) were born in Thailand, and over two-thirds (68.5%) had a birth certificate, including both those born in or outside of Thailand.

**Table 2:** Characteristics of the child, household, and primary carer and bivariate associations with the child’s health insurance status (*n*=803)

**Child’s, Household’s, and Primary carer’s characteristics**		**Total (*n*=803)**		**Health insurance status**				
				**Insured (*n*=135)**		**Uninsured (*n*=668)**		***p*-value**
		**%**	**(n)**	**%**	**(n)**	**%**	**(n)**	
**Socio-economic factors**								
Sex (MC)	Boy	49.7	(399)	51.1	(69)	49.4	(330)	0.717
	Girl	50.3	(404)	48.9	(66)	50.6	(338)	
Age (MC) *(median 7.0; IQR 7)*	0-5 years	36.5	(293)	31.1	(42)	37.6	(251)	0.044
	6 - 11 years	43.1	(346)	40.7	(55)	43.6	(291)	
	12 - 14 years	20.4	(164)	28.1	(38)	18.9	(126)	
HH income per head *(****median 1,667; IQR 1,389**** )*	Less than 1,000 Baht	18.6	(149)	23.0	(31)	17.7	(118)	0.225
	1,000-1,999 Baht	39.4	(316)	37.8	(51)	39.7	(265)	
	2,000-2,999 Baht	23.9	(192)	18.5	(25)	25.0	(167)	
	3,000 Baht and over	18.2	(146)	20.7	(28)	17.7	(118)	
Number of children in HH	1	16.8	(135)	15.6	(21)	17.1	(114)	0.763
	2	42.8	(344)	40.0	(54)	43.4	(290)	
	3	20.5	(165)	22.2	(30)	20.2	(135)	
	4 and more	19.8	(159)	22.2	(30)	19.3	(129)	
**Migration factors**								
Birthplace (MC)	In Thailand	76.2	(612)	86.7	(117)	74.1	(495)	0.001
	Not in Thailand	23.8	(191)	13.3	(18)	25.9	(173)	
Length of stay in Thailand (HH) *(me****dian**** 1****1****.****8; IQR**** 7.****8****)*	0-4 years	11.7	(94)	3.7	(5)	13.3	(89)	0.001
	5-9 years	27.6	(222)	25.2	(34)	28.1	(188)	
	10-14 years	29.1	(234)	26.7	(36)	29.6	(198)	
	15-19 years	17.2	(138)	23.0	(31)	16.0	(107)	
	20 years and over	14.3	(115)	21.5	(29)	12.9	(86)	
Return plan to home country (HH)	Within 2 years	4.1	(33)	2.2	(3)	4.5	(30)	0.263
	Within 2-5 years	11.1	(89)	7.4	(10)	11.8	(79)	
	No plan to return	48.4	(389)	51.9	(70)	47.8	(319)	
	No plan yet	36.4	(292)	38.5	(52)	35.9	(240)	
Future plan for the child (after 15 years old) (HH)	Not to be in Thailand	28.5	(229)	16.3	(22)	31.0	(207)	0.001
	To be in Thailand	71.5	(574)	83.7	(113)	69.0	(461)	
Work permit (PC)	Yes	38.1	(306)	40.7	(55)	37.6	(251)	0.490
	No	61.9	(497)	59.3	(80)	62.4	(417)	
**Health insurance-related factors**								
Birth registration (MC)	Not registered/ Not known	31.5	(253)	23.0	(31)	33.2	(222)	0.019
	Registered	68.5	(550)	77.0	(104)	66.8	(446)	
Health insurance status (PC)	Yes	59.3	(476)	32.6	(44)	64.7	(432)	0.000
	No	40.7	(327)	67.4	(91)	35.3	(236)	
Thai language ability (PC)	Good/very good	10.0	(80)	18.5	(25)	8.2	(55)	0.000
	Fair	16.3	(131)	25.9	(35)	14.4	(96)	
	Little/No	73.7	(592)	55.6	(75)	77.4	(517)	
Knowledge of MC’s right to health insurance (PC)	No	51.1	(410)	31.1	(42)	55.1	(368)	0.000
	Yes	48.9	(393)	68.9	(93)	44.9	(300)	
Total		100	(803)	100	(135)	100	(668)	

Note: *p-*values based on chi-square test of proportions

*MC* Migrant children, *HH* Household, *PC* Primary carer

Characteristics of the household of the migrant child indicate a median per capita monthly income of 1,667 baht and about two migrant children per household. Since first moving to Thailand, 29% of the families have spent 10-14 years in-country, while about 28% had spent 5-9 years. The median duration of stay in Thailand is 11.8 years. At the time of the survey, nearly half the sample had no plan to return to their country of origin, while one-third had not yet made a decision either way. Regarding plans for the migrant child when she or he reaches the age of 15 years, nearly three-fourths of the sample said they would like to see the child remain in Thailand and eventually get a job there.

Two-thirds of the primary carer of the migrant child did not have a work permit, but nearly 60% had health insurance. However, nearly three-fourths of these individuals only had limited ability or no ability at all to communicate in Thai. Less than half knew about the right of the migrant child to buy health insurance from the Thai government.

Comparing distributions of the sample of migrant children with health insurance and those without health insurance; classified by the characteristics of the child, the household of the child, and the primary carer; the bivariate analysis found that the health insurance status of the child was statistically significantly associated with the following variables: age and birthplace of the migrant child, length of stay in Thailand of the household, future plan for the migrant child of the household, and all of the health insurance-related factors, namely birth registration of the migrant child, health insurance status, Thai language ability, and knowledge about the child’s right to health insurance of the primary carer.

In the multivariate analysis, Model 3 of the Logistic GEE which entered all variables of socio-economic factors, migration factors, and health insurance-related factors into the analysis was confirmed to be the most suitable structure with the smallest statistics of QICC. Presented in Table 3, the significant predictors of whether or not the migrant child is covered by health insurance include the following variables: age of the migrant child (those age 12-14 years are more likely to be insured than children in other age groups, OR 2.34, *p*<0.01); future plan for the migrant child when she or he reaches age 15 (having a plan for the migrant child to remain in Thailand and seek employment at or after age 15 years was associated with greater likelihood that the child had health insurance, OR 2.37, *p*<0.05); work permit status of the primary carer (if the primary carer did not have a work permit, the migrant child was more likely to have health insurance, OR 4.12, *p*<0.01); birth registration status of the migrant child (children with a registered birth were more likely to be insured, OR 1.89, *p*<0.05), health insurance status of the primary carer (being insured was a predictor of the migrant child’s being insured as well, OR 8.51, *p*<0.01); Thai language ability (if the primary carer had little or no Thai language ability, then the migrant child was less likely to be insured, OR 0.31, *p*<0.01); and primary carer’s knowledge about the migrant child’s right to health insurance (if the primary carer was knowledgeable about the right of the child, the child was more likely to be insured, OR 2.57, *p*<0.01).

**Table 3:** Results of the Logistic GEE Analysis on the health insurance status of migrant children (*n*=803)

**Child, Household, and Primary carer factors**		**Model 1**			**Model 2**			**Model 3**		
		**Odds ratio**	**(95% CI)**		**Odds ratio**	**(95% CI)**		**Odds ratio**	**(95% CI)**	
			**lower - upper**			**lower - upper**			**lower - upper**	
**Socio-economic factors**										
Sex (MC)	Girl (ref - Boy)	0.95	(0.65-1.39)		0.91	(0.62-1.35)		0.86	(0.56-1.32)	
Age (MC)	0-5 years (ref.)	1.00			1.00			1.00		
	6 - 11 years	1.14	(0.74-1.74)		1.13	(0.74-1.75)		1.103	(0.68-1.78)	
	12 - 14 years	1.77^b^	(1.02-3.07)		2.05^b^	(1.12-3.74)		2.34^c^	(1.23-4.46)	
HH income per head	Less than 1,000 baht (ref.)	1.00			1.00			1.00		
	1,000-1,999 baht	0.78	(0.39-1.59)		0.79	(0.37-1.67)		1.09	(0.48-2.49)	
	2,000-2,999 baht	0.65	(0.29-1.45)		0.62	(0.28-1.38)		0.68	(0.24-1.95)	
	3,000 baht and over	1.06	(0.48-2.38)		0.95	(0.40-2.22)		1.10	(0.39-3.08)	
Number of children (0-14) in HH	1 (ref.)	1.00			1.00			1.00		
	2	0.97	(0.53-1.77)		0.89	(0.43-1.64)		0.91	(0.45-1.82)	
	3	1.16	(0.52-2.61)		1.23	(0.53-2.87)		1.10	(0.42-2.85)	
	4 and more	1.16	(0.53-2.53)		0.99	(0.43-2.27)		1.39	(0.53-3.64)	
**Migration factors**										
Birthplace (MC)	Not in Thailand (ref.- In Thailand)				0.43^b^	(0.20-0.92)		0.75	(0.35-1.59)	
Length of stay in Thailand	0-4 years (ref.)				1.00			1.00		
	5-9 years				2.47	(0.79-7.74)		2.64	(0.83-8.37)	
	10-14 years				1.81	(0.54-6.06)		1.38	(0.38-4.99)	
	15-19 years				2.61	(0.73-9.32)		2.28	(0.58-8.90)	
	20 years and over				2.69	(0.73-9.86)		1.47	(0.37-5.83)	
Return plan to home country	Within 2 years (ref.)				1.00			1.00		
	Within 2-5 years				1.03	(0.23-4.67)		1.49	(0.22-10.07)	
	No plan to return				1.11	(0.28-4.41)		1.11	(0.17-7.39)	
	No plan yet				1.23	(0.32-4.78)		1.25	(0.19-8.17)	
Future plan for the child (after 15 years old)	To be in Thailand (ref. - Not to be in Thailand)				1.88^a^	(0.94-3.75)		2.37^b^	(1.09-5.17)	
Work Permit (PC)	No (ref. – Yes)				0.85	(0.51-1.42)		4.12^c^	(1.88 – 9.06)	
**Health insurance-related factors**										
Birth registration (MC)	Registered (ref. - Not registered/ Not known)							1.89^b^	(1.04-3.45)	
Health insurance status (PC)	Yes (ref. -No)							8.51^c^	(3.93-18.41)	
Thai language ability (PC)	Good/very good (ref.)							1.00		
	Fair							0.74	(0.32-1.74)	
	Little/No							0.31^c^	(0.14-0.66)	
Knowledge of MC’s right to health insurance (PC)	Yes (ref. -No)							2.57^c^	(1.52-4.34)	
**Quasi Likelihood under Independence Model Criterion (QIC)**		746.96			743.68			646.94		
**Corrected Quasi Likelihood under Independence Model Criterion (QICC)**		736.86			723.49			626.10		

Note: ^a^significant at 0.1, ^b^at 0.05, ^c^at 0.01 levels

It is noteworthy that, while the birthplace of the migrant child was a significant predictor of health insurance status in Model 2, that relationship disappeared in Model 3, which introduced health insurance-related factors into the equation. The likely reason for that is the inclusion of the birth registration status of the child in Model 3, which is itself associated with birthplace. The descriptive analysis found that having a birth registration document was, to some extent, associated with the child’s birthplace. A higher percentage of children born in Thailand reported having a birth registration than those born in Myanmar. This finding also implies that access to health insurance for the migrant child is related to having a birth certificate or birth registration – regardless of the country of birth.

## Discussion

Regarding the findings, on socio-economic factors, it is not clear why children aged 12-14 years were more likely to have health insurance than children in the younger age groups. One hypothesis is that, when children grow up, especially those in school or migrant learning center, they are more likely to become knowledgeable about health insurance options and can educate their families about this. That hypothesis is consistent with the findings of other studies which found that level of education was associated with migrant child access to vaccination and other health services [15, 34, 40]. However, since the sample in this study included pre-school-age children (i.e., age 0-5), it was not appropriate to enter the school enrollment status variable in the multivariate models to examine whether it really explained. Still, it should be noted that it is the youngest children (age under 5 years) who are most vulnerable to morbidity and mortality and, thus, should be a priority for health insurance coverage.

On migration factors, whether the migrant household planned to have their child(ren) remain in Thailand (when they reached the age of 15 years) was a significant factor associated with health insurance coverage of migrant children. That plan might influence the parents or guardians to value health and access to health services of the child rather highly to build a quality adolescent and young adult. They know that if their child is ill or injured and cannot get the proper medical care, then that might have adverse consequences for the child over the longer-term remaining in Thailand. Such an adverse outcome would be a burden, not only for the child, but also for the family as well. Such long-lasting effects would also potentially limit the employment options in Thailand for the migrant child when they reach working age.

Another significant predictor is the work permit status of the primary carer. The findings for this variable are somewhat counter-intuitive. This study found that the primary carer’s not having a work permit was a significant predictor of the migrant child being covered by health insurance. One reason might be about the household’s financial constraints and expenses for a work permit that was already a big burden to the family which refrained them from purchasing health insurance for the child [18]. Work permit’s related expenses including fees for temporary residence permit, medical checkups, health insurance, and extra costs of transportation and, in many cases, broker service fees could cost as high as 8,000-10,000 baht to migrant workers. Another point is that the migrant parent who had a legal work permit might be at work and away from home for much of the day which meant that the care for the migrant child was less close or of lower quality than parent/guardian who was not working or without a work permit [5]. This suggests that - with support from relevant stakeholders as a workplace-based intervention - all workplaces with non-Thai migrant workers should offer pathways to affordable health insurance for the workers themselves as well as their accompanying dependents. However, the association between the carer’s work permit status and the child’s health insurance status needs qualitative information to analyze more in-depth and confirm the explanation.

In terms of the health insurance-related factors, having a birth registration document increased the likelihood that the migrant child would be covered by health insurance. The birth documentation might be from the home country or Thailand to have this effect. The birth registration is a key identity document for the child and has equal importance as a passport to establish citizenship. That, in turn, opens up access to all the basic rights that the child should access, no matter where they live (Ensor & Gozdziak, 2010 cited in [41]). This is especially important for primary education and early childhood health promotion and prevention. A previous qualitative study in Thailand found that a birth registration document was a crucial condition for cross-border migrants who wanted to purchase a health insurance card (the MHIS of the MOPH) for their accompanying dependents. Even though the MHIS does not require full documentation of the person, many of the public hospitals which administer the enrollment have requested that the applicant show a valid personal identification document (which, in the case of the child migrant, would be a birth registration) as a condition for purchasing the health insurance card [14].

The health insurance status of the primary carer was one of the insurance-related factors that increase access to health insurance for the migrant child [35]. When the primary carer has taken no trouble to obtain health insurance for themselves, then it is logical that they would also seek it for the dependent child. That is because they already have the knowledge, understanding, and motivation for getting health insurance as a basic need for living in Thailand, not only for themselves but also the accompanying dependents. Similarly, the carer’s Thai language ability had a positive association with health insurance coverage for the migrant child. This is also logical in that it would normally take some ability to read and understand the Thai language to be fully informed about health insurance rights and options, and the procedure for obtaining coverage [42]. Even though many of the public health outlets at border areas have bi-lingual Migrant Health Workers (MHW) who can help translate and interpret, there is still a limited number of these MHW [12]. Knowing the right that every migrant child in Thailand, regardless of documentation status, can purchase a health insurance card with the MHIS is another important enhancing factor. Primary carers who did not have this essential knowledge were less likely to be caring for a migrant child who is covered by health insurance.

## Conclusions

This study had the objective to analyze factors associated with the health insurance status of migrant children, aged 0-14 years, who were born or accompanied their migrant parent(s) to live in Thailand. Three groups of independent variables were entered into the analysis which reflect the socio-economic factors, migration factors, and health insurance-related factors. Overall, the insurance-related factors tended to be the strongest factor associated with health insurance status for the migrant children, especially the characteristics of their primary carer. Almost important were the migration status of the primary carer, and whether she or he had a work permit. Somewhat counterintuitively, those children whose primary carer did not have a work permit were more likely to have health insurance. The family’s future plan for the child to remain in Thailand after reaching the age of 15 years was also associated with having health insurance coverage. Access to or having health insurance for the child was correlated with the age of the child and having a personal identification document, i.e., birth registration. The age of the migrant child was the only socio-economic factor that was significantly associated with the health insurance status of the child. These findings would be of great benefit in exploring ways to promote access to health protection for the cross-border migrant children while living in Thailand.

This study has some limitations worth mentioning. Firstly, some standard factors of enrolment and access to health insurance of the child (e.g. perception of migrants on price and cost-benefit of having health insurance, service quality, and other supply-side factors) are not included in the analysis. Secondly, the study used cross-sectional data which by nature the cause-and-effect relationship cannot be fully inferred. Thirdly, though the sample size is large, the samples were not randomly selected, thus, they cannot represent all migrant children in the study areas. Lastly, sub-samples analyses of migrant children who were covered by different types of health insurance (non-governmental, e.g. M-Fund, and governmental ones, the MHIS) were not conducted. This was due to too small number of sub-samples of migrant children under each health insurance.

Based on the findings, recommendations to improve access to health insurance for migrant children in Thailand are as follows. Firstly, higher priority should be given to increasing access to birth registration for all children born in the country regardless of their parents’ legal statuses. Secondly, there should be increased motivation and education of migrant workers who have accompanying dependent children about child rights and the importance of insurance, especially those with an early child aged 0-5 years. Thirdly, there should be more programs and supports to reduce the language barrier faced by the migrant population in accessing the relevant information and processes to purchase health insurance for their accompanying child(ren). Fourthly, there should be more information dissemination about the health insurance options for migrants with accompanying children through workplace-based interventions.

The protection of human rights including the right to health for all children is in accordance with the principles of the Convention on the Rights of the Child (CRC) which Thailand signed in 1992. In addition to improving health insurance coverage for migrant children, it would be better if there the country implement a policy to provide free basic healthcare including health promotion and disease prevention services to all children, regardless of their nationality and insurance status.

## Supplementary Information

**Additional file 1. **Questionnaire (Questionnaire of the research project “Migrant children population: Child rearing, access to health services and education in Special Economic Zone (SEZ) Mae Sot, Tak Province”).

## Data Availability

The datasets generated and/or analyzed during the current study are not publicly available because it was obtained from a questionnaire survey with cross-border migrant parents who are considered vulnerable in many aspects - especially those who were of undocumented status - but are available from the corresponding author on reasonable request.

## Abbreviations

CI: Confidence Interval
CRC: Convention on the Rights of the Child
GEE: Generalized Estimating Equation
HH: Household
IOM: International Organization for Migration
MC: Migrant Children
M-Fund: Migrant Fund
MHIS: Migrant Health Insurance Scheme
MHW: Migrant Health Workers
MOPH: Ministry of Public Health
OR: Odds ratio
PC: Primary Carer
QICC: Quasi Likelihood under Independence Model Criterion
Ref.: Reference group
UNICEF: United Nations Children's Fund

## Footnotes

1 Unfortunately, some standard factors of enrolment and access to health insurance (especially the supply-side factors) are not included due to limitations of data collection and the cross-sectional study design that focuses on the factors of migrant children, their household, and the primary carer.

2 The formula is “n = N/(1+N*e^2^)”; where “n” is the sample size, “N” is the population size and “e” is the margin of error.
