# Supplementary material for: Health insurance status of cross-border migrant children and the associated factors: a study in a Thai-Myanmar border area
Source: BMC Health Serv Res. 2022 Oct 23;22:1276. doi: 10.1186/s12913-022-08681-0 (PMC9590151; doi:10.1186/s12913-022-08681-0)
Supplement: Supplementary file 1 — Additional file 1. Questionnaire (Questionnaire of the research project “Migrant children population: Child rearing, access to health services and education in Special Economic Zone (SEZ) Mae Sot, Tak Province”). [file 12913_2022_8681_MOESM1_ESM.docx]

**Questionnaire:**

**“Migrant children population: Child rearing, access to health services and education in Special Economic Zone (SEZ) Mae Sot, Tak Province”**

**Part 1: Basic Information on Household member:**

| 1.1  Member No. | 1.2  **First/Last**  **Name**  (Member who  lived in this  household for at least 3 month ) | 1.3 **Sex**  1. Male  2. Female | 1.4  **Age**  (years) | 1.5**.1**  **Education**  **/Learning development**  1.Never enrolled  2.Ever enrolled  3. Studying/enrolling | **1.5.2 Highest level**  **of education/**  **Learning development**  (See codes) | 1.6 **Marital**  **status**  1. Single  2. Married  3. Widowed  4. Divorced  5. Separated | 1.7 **Occupation**  What does this person do?  (type of job)  (See codes) | **1.8 Having non-expired document that allowed to stay in Thailand**  (See codes) | **1.9 Having a non-expired work permit?**   1. Yes 2. No 3. Ever had | **1.10 Having non-expired health insurance/security?**  (See codes) | **1.11 Thai Language ability (overall)**   - 1. Very good   2. Good   3. Sufficient   4. Little   5. Not at all | **1.12 Relationship**  **with head of**  **household**  (See codes) |
| --- | --- | --- | --- | --- | --- | --- | --- | --- | --- | --- | --- | --- |
| 1. |  |  |  |  |  |  |  |  |  |  |  |  |
| 2. |  |  |  |  |  |  |  |  |  |  |  |  |
| 3. |  |  |  |  |  |  |  |  |  |  |  |  |
| 4. |  |  |  |  |  |  |  |  |  |  |  |  |
| 5. |  |  |  |  |  |  |  |  |  |  |  |  |
| 6. |  |  |  |  |  |  |  |  |  |  |  |  |
| 7. |  |  |  |  |  |  |  |  |  |  |  |  |
| 8. |  |  |  |  |  |  |  |  |  |  |  |  |
| 9. |  |  |  |  |  |  |  |  |  |  |  |  |
| 10. |  |  |  |  |  |  |  |  |  |  |  |  |

Q1.13 When did your household first move to Thailand?

……………. Month………………Year 2. Don't remember 3. No response

Q1.14 Does the household have any plan to return home in Myanmar (not short term visit but long term or permanent)?

1. This year 2. 1-2 years later 3. 3-5 years later 4. No plan 5. No plan YET 6. Don’t know/remember 7. No response

Q1.15 How much on average per month of your household income (from all members)?

_________________ Baht/ Month (or ______________________________Baht/Year)

**Codes for 1.5.2 Education/Learning development**

1. No education 2. Learning Center, Nursery 3.Primary school (Myanmar) 4. Middle school (Myanmar)

5. High school (Myanmar) 6. Primary school (Thai) 7. Junior high school (Thai) 8. High school/vocational school (Thai)

9. Diploma/High vocational certificate 10. Bachelor’s degree 11.Other ……………..……… 12. Don’t know

**Codes for 1.7 Occupation**

1. Working (with income)/Employed 2. Waiting for Seasonal Work 3. Unemployed/ Looking for work

4. Retired/Too old 5. Long-term illness and disabilities 6. Caring for other HH members

7. Going to school 8. Not working 9. Other (Specify)…………..

**Codes for 1.8 Document**

1. No Document 2. Passport 3. Temporary passport/CI (from NV) 4. Registration Card

5. Tor.Ror 38/1 6. Pink Card (2 years) 7.Pink Card (5 years ) 8. White Card (10 years )

9. Expired document, identify…………………………. 10. Other…………………… 11.Don’t know

**Codes for 1.10 Health insurance/security**

1. Migrant health insurance Card (age 7 and over) 2, Migrant health insurance Card (age lower than 7) 3. Social Security system

4. Private health insurance 5. Other (specify)………….. 6. Do not have

7. Don’t know 8. No response

**Codes for 1.12 Relationship with head of household**

1. Head of household 2. Spouse 3. Father 4. Mother

5. Father/mother-in-laws 6. Son/daughter 7. Sibling 8. Son-in-law

9. Employee 10. Brother/sister 11. Daughter-in-law 12. Great grandchild

13. Nephew/niece 14. Friend 15. Relative 16. Other (Specify)………

**Part 2: Information of household members aged 0-14 years**

| Member No. (Copy from Q.1.1) | **Father**  If in the same household, copy member No. from Q1.1.)  If not in the household,  21. in Thailand  22. in Myanmar  23. other country  24. Dead  25. Do not know. | **Mother**  If in the same household, copy member No. from Q1.1.)  If not in the household,  21. in Thailand  22. in Myanmar  23. other country  24. Dead  25. Do not know. | **2.1**  **Where were children born?**  1. Thai (to Q2.3)  2. Not in Thai (to Q2.2) | **2.2 If not in Thai, was the child registered for birth registration?**  1. Yes  2. No  3. Don’t know  (skip to Q2.8) | **2.3**  **If in Thai, Where were the child delivered?**  (See codes) | **2.4 If in Thai, Was the child received birth certificate (usually, from the health facility where the delivery was taken place) ?**  1. Yes  2. No  3. Don’t know | **2.5**  **If in Thai, Was the child registered for birth registration?**  1. Yes  2. No (skip to Q2.7)  3. Don’t know | **2.6 If yes, by whom?** (a person who proceeded document at the Civil Registration office) (Multiple answers are allowed)  (See codes) | **2.7 If not, why?**  (See codes) | **2.8 Is the child studying/enrolling in a learning center?**  1. Yes  2. No | **2.9**  **If yes, what type of school?**  1.Thai/regular school  2. Thai/non-regular school  3. NGO school/Learning center  4. Other… | **2.10 After age 14 (age 15 and over),** where does the household plan for the child to live?  1.In Thailand, Mae Sot  2. In Thailand, other places  3.In Myanmar  4.other countries  5. Do not plan yet/Do not know  6. No response |
| --- | --- | --- | --- | --- | --- | --- | --- | --- | --- | --- | --- | --- |
|  |  |  |  |  |  |  |  |  |  |  |  |  |
|  |  |  |  |  |  |  |  |  |  |  |  |  |
|  |  |  |  |  |  |  |  |  |  |  |  |  |
|  |  |  |  |  |  |  |  |  |  |  |  |  |
|  |  |  |  |  |  |  |  |  |  |  |  |  |

**Code for 2.3**

1. At home or in the community 2. Public hospital 3. Private hospital

4. Health center 5. NGO’s health facility 6. Other…………….………..

**Code for 2.6**

1. Parents (father or mother) 2. Grandparent 3. Relatives

4. Friends/colleagues of parents 5. Hospital staff 6. Community leader

7. NGO 8. Other…………………

**Code for 2.7**

1. Registration place was too far 2. Parents are not registered/undocumented 3. No transport/inconvenient transportation

4. No money 5. Inconvenient opening hours 6. Lack of personnel assisting for registration

7. Poor quality of registration services/bad experiences 8. Don’t know the places 9. Cannot speak Thai

10. Not necessary 11. Don’t know registration procedure 12. Don’t know 13.Other ………………………

**Part 3: Characteristics; knowledge and attitude about child rights of the parents/guardian of the member aged 0-14 years**

| Member No. (Copy from Q.1.1) | | _______________________________ | | |
| --- | --- | --- | --- | --- |
| Q 3.1 | What ethnic group do you belong to? |  |  |  |
| Q 3.2 | How long have you lived in Thailand ?  *(Since first time you have come to Thailand)* | ……………..…….…… Year  …………………….….. Month  Don’t remember |  |  |
| Q3.3 | What is your main occupation in Thailand now? |  |  |  |
| Q3.4 | How much on average per month of your income (from all sources)? | _________________Baht/month |  |  |

|  | Do you know that? | Yes | No | Do not know |
| --- | --- | --- | --- | --- |
| Q3.5 | All children born in Thailand, regardless of legal status of parents, are eligible to be registered and received a birth registration | 1 | 2 | 3 |
| Q3.6 | All children - regardless of the nationality or legal status - living in Thailand are eligible to basic education provided by Thai government | 1 | 2 | 3 |
| Q3.7 | Migrant children aged less than 7 are eligible to buy a health insurance card at price 365 Baht/Year at Thai Public Hospital | 1 | 2 | 3 |
| Q3.8 | Migrants aged 7 and over are eligible to buy a health insurance card at price 1,600 Baht/Year at Thai Public Hospital | 1 | 2 | 3 |
| Q3.9 | Migrant workers with a passport/or whose Nationality Verified and a work permit are eligible to register with the Social Security Scheme | 1 | 2 | 3 |

|  | Do you think that, in practice? | Definitely yes | Probably Yes | Probably No. | Definitely No |
| --- | --- | --- | --- | --- | --- |
| Q3.10 | All children born in Thailand, regardless of legal status of parents, can access to registration and receive a birth registration? | 1 | 2 | 3 | 4 |
| Q3.11 | All children - regardless of the nationality or legal status - living in Thailand can access to basic education provided by Thai government | 1 | 2 | 3 | 4 |
| Q3.12 | Migrant children aged less than 7 can access to buy a health insurance card at price 365 Baht/Year at Thai Public Hospital? | 1 | 2 | 3 | 4 |
| Q3.13 | Migrants aged 7 and over can access a health insurance card at price 1,600 Baht/Year at Thai Public Hospital? | 1 | 2 | 3 | 4 |
| Q3.14 | Migrant workers with a passport/or whose Nationality Verified and a work permit can access to register with the Social Security Scheme? | 1 | 2 | 3 | 4 |
